# Multiplexing clonality: combining RGB marking and genetic barcoding

**DOI:** 10.1093/nar/gku081

**Published:** 2014-01-28

**Authors:** Kerstin Cornils, Lars Thielecke, Svenja Hüser, Michael Forgber, Michael Thomaschewski, Nadja Kleist, Kais Hussein, Kristoffer Riecken, Tassilo Volz, Sebastian Gerdes, Ingmar Glauche, Andreas Dahl, Maura Dandri, Ingo Roeder, Boris Fehse

**Affiliations:** ^1^Research Department Cell and Gene Therapy, Department of Stem Cell Transplantation, University Medical Centre Hamburg-Eppendorf, Martinistrasse 52, Hamburg 20246, Germany, ^2^Institute for Medical Informatics and Biometry, Faculty of Medicine, Technische Universität Dresden, Dresden 01307, Germany, ^3^ALS Automated Lab Solutions GmbH, Jena 07747, Germany, ^4^Department of Neuropathology, Hannover Medical School, Institute of Pathology, Hannover 30625, Germany, ^5^Department of Internal Medicine I, University Medical Center Hamburg-Eppendorf, Hamburg 20246, Germany, ^6^Deep Sequencing Group SFB 655, Biotechnology Center, Technische Universität Dresden, Dresden 01307, Germany

## Abstract

RGB marking and DNA barcoding are two cutting-edge technologies in the field of clonal cell marking. To combine the virtues of both approaches, we equipped LeGO vectors encoding red, green or blue fluorescent proteins with complex DNA barcodes carrying color-specific signatures. For these vectors, we generated highly complex plasmid libraries that were used for the production of barcoded lentiviral vector particles. In proof-of-principle experiments, we used barcoded vectors for RGB marking of cell lines and primary murine hepatocytes. We applied single-cell polymerase chain reaction to decipher barcode signatures of individual RGB-marked cells expressing defined color hues. This enabled us to prove clonal identity of cells with one and the same RGB color. Also, we made use of barcoded vectors to investigate clonal development of leukemia induced by ectopic oncogene expression in murine hematopoietic cells. In conclusion, by combining RGB marking and DNA barcoding, we have established a novel technique for the unambiguous genetic marking of individual cells in the context of normal regeneration as well as malignant outgrowth. Moreover, the introduction of color-specific signatures in barcodes will facilitate studies on the impact of different variables (e.g. vector type, transgenes, culture conditions) in the context of competitive repopulation studies.

## INTRODUCTION

Permanent cell marking by integrating (retroviral) vectors has been used to track cell populations or even single cells *in vitro* and *in vivo* (1). Cell marking studies have provided important insights into biology and development of cells, tissues, organs and even whole organisms (2). Moreover, for many years, gene marking has been considered one of the most successful approaches in human gene therapy (3).

The cloning and successful expression of *Aequorea victoria* green fluorescent protein (GFP), first described in the 1970s, facilitated direct visualization of gene-marked cells and thus initiated a new boost of marking approaches in experimental biology and biomedicine (2,4). Based on the subsequent cloning of further fluorescent proteins, interactions of differently labeled cell populations could be studied (5). Recently, multi-color marking techniques have been introduced based on complex recombination strategies (‘Brainbow’ imaging) (6) or simultaneous transduction with different lentiviral vectors (‘RGB marking’) (7) that allow for the phenotype-based identification of differently marked cells down to the clonal level.

Alternative strategies to monitor individual cell clones rely on molecular methods. A technique broadly applied in experimental, but also clinical, settings makes use of the unique vector integration sites (VISs) in the target cell genome characteristic for retroviral vectors. After mapping a VIS in the host cell genome, VIS-specific quantitative polymerase chain reactions (PCRs) can be used to assess a clone’s contribution, e.g. to hematopoiesis over time (8). Alternatively, methods for high-throughput retrieval of insertions sites, such as ligation-mediated (LM) and linear-amplification-mediated PCR can be directly combined with next-generation sequencing (NGS) techniques for large-scale assessment and quantification of insertion sites (9,10). However, linear amplification-mediated PCR has been associated with significant biases resulting in the selective amplification of some insertion sites and loss of others (11,12). To overcome this limitation, the introduction of short DNA tags termed ‘barcodes’ into cell genomes has been suggested as a novel means for cell marking (13–15). To this end, integrating vectors were equipped with short, highly variable DNA sequences that allow unequivocal identification of individually marked cells [reviewed by Bystrykh *et al.* (16)]. Given that several preconditions such as sufficient complexity of the barcode library are met (16), barcode marking should allow unbiased and precise analyses of quantitative contributions of marked cells to any population of interest.

As single strategies, both phenotypic and genetic clonal marking have their advantages and limitations. Phenotypic marking allows for visualization of cells in their natural context, but relies on constant transgene expression; genetic marking has a high resolution power and is independent of expression, but requires tissue destruction. Therefore, we here propose to combine the advantages of both techniques by introducing specific barcodes equipped with color-specific signatures into our LeGO vectors (17) previously shown to facilitate RGB marking (7). We also developed barcoded LeGO-IRES vectors for simultaneous expression of a gene-of-interest and a fluorescent marker protein for the analysis of gene functions. In proof-of-principle experiments, we show that fluorescent cell marking with barcoded LeGO vectors facilitates clonal analysis both *in vitro* and *in vivo*, in models of normal tissue regeneration as well as malignant outgrowth. Clonal identity could be confirmed at two different levels—*in situ* based on fluorescent microscopy and *in silico* based on sequenced barcodes.

## MATERIALS AND METHODS

### Generation of barcoded LeGO-vector libraries

For introduction of the barcode sequence, the original LeGO-vectors [LeGO-V2, -Cer2, -C2, -G2 and -iG2 (17)] were equipped with a dedicated barcode cloning site containing the unique restriction enzyme recognition sites for XbaI und XhoI. Color-specific barcodes containing 16 randomized nucleotides (BC16, see below) were generated by annealing complementary forward and reverse oligonucleotides manufactured by TIB Molbiol. Fifty picomoles of each strand were mixed in 500 mM Tris–HCl (pH 7.6), 100 mM MgCl_2_, 50 mM dithiothreitol and 1 mM spermidine and annealed under the following conditions: starting from 95°C, the temperature was lowered to 75°C in steps of 1°C after 10 min of incubation. From 74 to 22°C the temperature decreased in 1°C steps after incubation of 1 min. Hybridized oligonucleotides that already contained restriction-site-specific overhangs were afterward phosphorylated and ligated in 50-fold excess into the respective XbaI/XhoI-digested LeGO vector backbone. After dialysis on 0.025 -µm MF-Millipore-filter, the whole ligation reaction was transformed into 40 µl of MegaX DH10B electrocompetent cells (Life Technologies) under the following conditions: 1.8 kV, 200 Ω and 25 µF. An aliquot of the transformation reaction (0.1%) was plated on agar plates for counting colonies to estimate the theoretical complexity of the barcode library.

### Sequencing to assess for library complexity (plasmid bulk)

In all, 10^10^ copies of the plasmid preparation were used for amplification of a 229-bp barcode-containing fragment in a 40-cycle PCR reaction using the Multiplex PCR Plus Kit (Qiagen) according to manufacturer’s protocol, with 57°C as annealing temperature (primers used: BC-PCR-FW and BC-PCR-RV_neu, see Supplementary Table S1, all primers were from Eurofins MWG Operon). PCR products were purified with Agencourt AMPure XP-beads (Beckman Coulter). To attach Illumina adaptors to the PCR products, a tailing PCR was performed with 25 cycles using the Multiplex PCR Plus Kit with 40°C as annealing temperature (primers: Ill1-Tail12 and Ill2_Tail-complete). PCR products were purified with Agencourt AMPure XP-beads afterward. Two microliters of PCR fragments was used for final construction of the indexed Illumina sequencing libraries. A tailed PCR using Illumina indexing primers was performed in 10 µl containing 1× Phusion High Fidelity Mix (NEB), 0.4 U Phusion polymerase (NEB), 5 pmol of a universal primer (P34), an indexing primer and 0.1 pmol of a bridging oligonucleotide. Sequencing was performed on a HiSeq 2000 system (Illumina).

### Quality criteria

The barcode design is based on an alternating sequence of pairs and triplets of random and nonrandom nucleotides (14). To separate true barcode sequences from false positives within the NGS results, only sequences that matched perfectly at all 22 nonrandom barcode positions and with a frequency of at least 10 were included in the analysis.

### Production of viral supernatant, titration and transduction of HEK293T cells

Production of viral supernatants and titration were performed as described earlier (17,18). For generating RGB-marked 293 T cells, 500 000 cells were seeded in six-well plates and transduced with the three different LeGO-BC16 vectors at multiplicities of infection (MOIs) of 1.4 for each vector.

### Single-cell picking with CellCelector

In all, 50 000 RGB-marked 293 T cells were seeded per well of a six-well plate. After 4 days of incubation, single cells were picked from colonies of the same color by using the CellCelector (ALS). In brief, cells of interest were chosen microscopically on a life image screen by mouse click. Selected single cells were automatically picked with an aspiration volume of 50 nl using a glass capillary with a diameter of 30 µm. Right before cell picking the glass capillary took up 2 µl of lysis buffer without Proteinase K (19). Each picked single cell was then transferred into the lid of a 0.2-ml PCR tube preloaded with a drop of 8 µl of lysis buffer.

### Single-cell PCR and barcode calling

Picked cells were lysed in a buffer containing 50 mM Tris (pH 8.0), 10 mM ethylenediaminetetraacetic acid, 100 mM NaCl and 200 µg/ml of Proteinase K for 1 h at 37°C (19). After inactivation of the enzyme, the whole batch was used to amplify the barcode sequences in a nested PCR procedure. First PCR using primers p90 and p91 resulted in a ca. 700-bp fragment. Five microliters from a 1:20 dilution served as the template for the nested PCR (primers: BC-PCR-FW and BC-PCR-RV_neu) in a 30-cycles reaction using the Multiplex PCR Plus Kit (Qiagen) according to the manufacturer’s protocol. The obtained PCR product was analyzed on a 1.5% agarose gel; the 229-bp fragment was subcloned into TOPO vector (Life Technologies) after purification with a gel extraction kit (Qiagen). Ten subcloned colonies were picked, and directly transferred into PCR mixes for amplification of the barcode sequence using the following protocol: 0.4 µM BC-PCR-FW- and BC-PCR-RV_neu-primer were used with the ReddyMix Master Mix (Thermo Scientific) with an annealing temperature of 57°C according to the manufacturer’s protocol. Five microliters of the PCR reaction was analyzed on a 1.5% agarose gel, and the remaining 20 µl was purified and directly sequenced using the BC-PCR-Seq primer (Seqlab).

### Transduction of primary hepatocytes and transplantation

Primary hepatocytes from C57Bl/6 J mice were isolated and cultured as previously described (7). Freshly prepared cells were transduced with the LeGO-C2-BC16 (red), LeGO-V2-BC16 (yellow-green) and LeGO-Cer2-BC16 (cyan-blue) at equal MOIs of 80 for 45 min at 37°C. In all, 1 × 10^6^ cells were transplanted intrasplenically into hemizygous uPA–SCID mice anesthetized with isofluoran (7). Mice were sacrificed 4 weeks after transplantation. The liver was fixed in 4% PFA for 4 h, dehydrated in 20% sucrose and embedded in Tissue-Tek (Sakura) for cryopreservation.

### Laser microdissection

Laser microdissection of single cells was performed as described by Hussein (20). In brief, 6 µm cryosections of the reconstituted liver were transferred onto lysine-coated ultraviolet-light-treated membrane slides (MMI) and put on a fluorescence microscope with a SmartCut Plus laser microdissection device (MMI). The area of interest was marked on a fluorescence microscopy image of a sequential section and was identified directly by fluorescence microscopy on the membrane slide, which was used for laser microdissection. For single-cell isolation, the lid of a 0.5-ml tube was pressed on the membrane and had no contact with the tissue because the tissue is on the opposite surface and the cut single cell sticks to the adhesive inlay of the lid. Cell lyses with 5 µl of Proteinase K buffer and amplification of barcode sequences were carried out as described before.

### Induction of leukemia

The ΔTrkA transgene was cloned into LeGO-iG2 equipped with unique XbaI and XhoI restriction sites (see above); barcodes were introduced as described above. As control, we used the barcoded LeGO-G2 marking vector (Supplementary Figure S1a). The resulting plasmid libraries were used to produce eco-pseudotyped viral supernatant. Lineage-negative (lin^−^) bone marrow cells from male donors were transduced with LeGO-ΔTrkA-iG2-BC16 (MOI: 4), or LeGO-G2-BC16 (MOI: 5) on Retronectin-coated plates. Four female recipient mice (Balb/C) per group were transplanted with 300.000 cells per mouse after total body irradiation at 8.5 Gy. Blood sampling was performed every 4 weeks, and eGFP expression and B220-positivity (B220-antibodies from BD Biosciences) in peripheral blood cells were measured by flow cytometry. Genomic DNA was isolated from hematopoietic cells and used for amplification of barcode sequences as well as NGS. DNA from spleen cells was also used for LM-PCR to identify integration sites of barcode vectors and for vector copy number determinations by digital droplet PCR (ddPCR).

### LM-PCR and ddPCR

LM-PCR to retrieve vector insertion sites was performed as previously described (21) using the primers for lentiviral vectors described in (22). To determine copy numbers of integrated vectors, we performed ddPCR. In a duplex reaction, a vector-specific fragment (using primers FP-dPCR-fw and FP-dPCR-rv primers and the FAM-labeled FP-probe) and a control amplicon (located in the erythropoietin receptor, using primers mEpo-fw and mEpo-rv and the HEX-labeled mEpo-probe) were simultaneously amplified. In all, 200 ng of genomic DNA was used as a template with 900 nMol of each primer and 250 nMol of each probe in the 2× ddPCR Supermix for probes (BioRad); 40 cycles of PCR were performed according to the protocol. Droplets were generated and analyzed using the QX100 system (BioRad).

## RESULTS

### Generation of barcoded vector libraries

**Figure 1.**
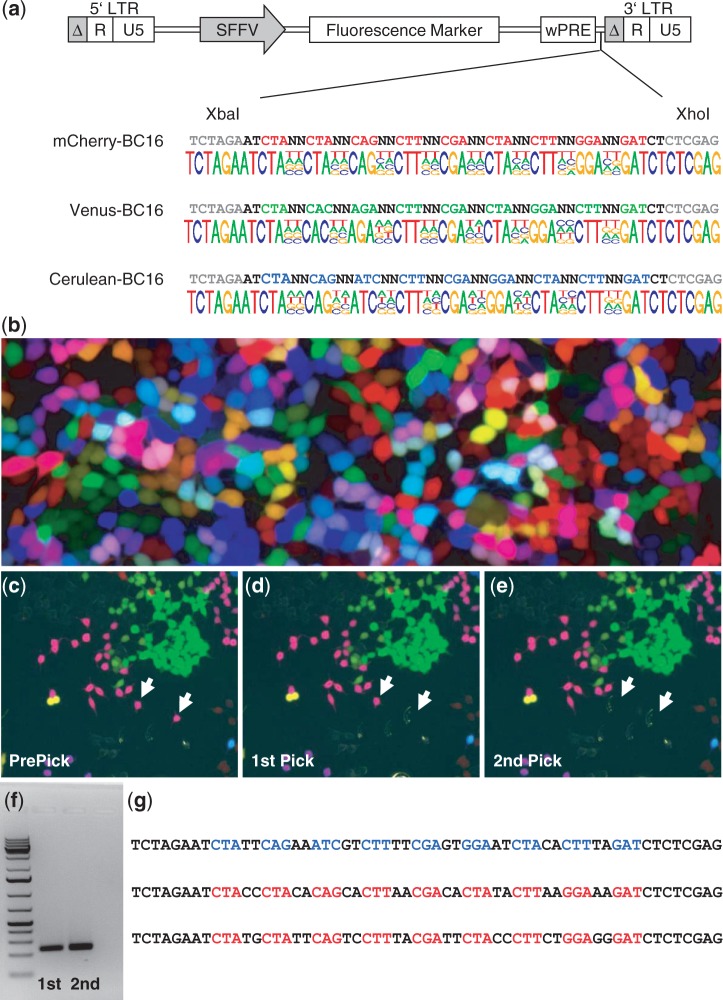
Vector construction and deciphering the color-code by single-cell PCR. (**a**) Barcodes with color signatures were introduced in front of the 3′ LTR of the respective LeGO vector by recombinant DNA technology. The barcodes consist of 16 random nucleotides separated by fixed triplets, the pattern of triplets cipher for the respective fluorescent protein in the vector. In all, 10^10^ plasmids of each plasmid library were used for NGS via Hiseq 2000 (Illumina). For all three RGB vectors, even distribution of the 4 nt reflecting comprehensive randomization during synthesis was found (based on at least 25 million reads for each vector). (**b**) For RGB transduction of HEK293T cells, equal amounts of viral particles of the LeGO-C2-BC16 (red), LeGO-V2-BC16 (yellow-green) and LeGO-Cer2-BC16 (cyan-blue) were used. (**c–e**) Two identically colored single cells from a pink colony were consecutively picked with the CellCelector. (**f**) Single-cell PCR generates a barcode-containing fragment of 229 bp for both cells picked in (d) and (e). (**g**) After subcloning of the PCR fragment into plasmids, sequencing of individual clones revealed a barcode signature of one blue and two red viral copies in each of the picked cells, which is in good agreement with the pink color of the analyzed clone following the additive color model.

We first generated four different barcodes for LeGO vectors encoding the fluorescent proteins mCherry (red), Venus (yellow-green), Cerulean (cyan-blue) (Figure 1a) and eGFP (green) (Figure 3a and Supplementary Figure S1a). All barcodes consisted of eight pairs of random nucleotides intersected by triplets of fixed nucleotides. This design (14) was chosen to avoid accidental generation of restriction enzyme recognition sites and/or sequence homologies resulting in secondary structure formation. At the same time, we used the fixed sequences to equip the different barcodes with a color-specific signature. This allows determining the vector of origin for any barcode being retrieved in later functional studies.

To generate double-stranded DNA barcodes, we made use of complementary single-stranded DNA oligonucleotides with 16 random positions (Supplementary Table S1 for details) corresponding to a maximum theoretical complexity of 4^16 ^= 4.29 × 10^9^. We ligated the short double-stranded DNA fragments by directed cloning into the restriction sites XbaI and XhoI previously introduced into the aforementioned three RGB vectors and two GFP vectors, namely, LeGO-V2-BC16, LeGO-C2-BC16, LeGO-Cer2-BC16, LeGO-G2-BC16 and LeGO-ΔTrkA-iG2-BC16. Using the ligation mixes, we generated plasmid libraries for all five barcoded vectors; the actual complexities of the libraries, as assessed based on bacteria-colony counts were in the range of 4 − 5 × 10^5^ for the four individual vectors.

### Sequence analysis of the plasmid libraries

For all vectors, we used NGS to assess the quality of generated barcodes. We used 80 pg DNA corresponding to 10^10^ plasmid copies as PCR template for barcode amplification. A mean of 33 million sequence reads (range: 25–43 Mio) were obtained, of which ∼80% passed quality criteria (not shown). As illustrated in Figures 1a and 3a and Supplementary Figure S1a, we found essentially equal frequencies (∼25%) of the 4 nt at all 16 random positions. This ensures a maximal nucleotide dissimilarity between the individual barcodes in the library [assessed by their Hamming distance (23)] and thus allows for their efficient distinction and error correction during later bioinformatics analysis.

We used the same sequencing data to validate the complexity of the plasmid library. After exclusion of barcode sequences with a low frequency (<10, to minimize the proportion of false-positive barcodes due to sequence errors in PCR and NGS) ∼5 × 10^5^ unique barcodes were found for each barcode (Venus: 4.76 × 10^5^, Cherry: 7.57 × 10^5^, Cerulean: 5.79 × 10^5^, eGFP: 5.74 × 10^5^, ΔTrkA-GFP: 7.32 × 10^5^). This is in good agreement with the predictions based on counted bacteria colonies (see above).

### Barcode-RGB marking and deciphering the color code by single-cell PCR

In the next step, we used the barcoded vectors LeGO-C2-BC16 (red), LeGO-V2-BC16 (yellow-green) and LeGO-Cer2-BC16 (cyan-blue) for RGB marking of 293 T cells (Figure 1b). RGB-marked cells were seeded at low cell density to allow outgrowth of identically colored clones (7). To proof identity of cells presenting themselves with the same pink color hue (Figure 1c), we picked two single cells using the CellCelector system (Figure 1d and e). We performed single-cell PCR to amplify barcode-containing fragments (Figure 1f) and cloned the PCR products into TOPO vectors. Sequencing of single bacteria clones revealed the presence of three different barcodes (Figure 1g) for the two cells shown in Figure 1c–e. Two barcodes represented the ‘red’ LeGO-C2-BC16, and one the ‘cyan-blue’ LeGO-Cer2-BC16; the pink color of the analyzed clone (Figure 1c–e) is thus in perfect agreement with the additive color model. Importantly, the combination of the three mentioned barcodes (Figure 1g) was found independently in each picked single cell (Figure 1d and e).

### Analyzing single cells from regenerated liver tissue

**Figure 2.**
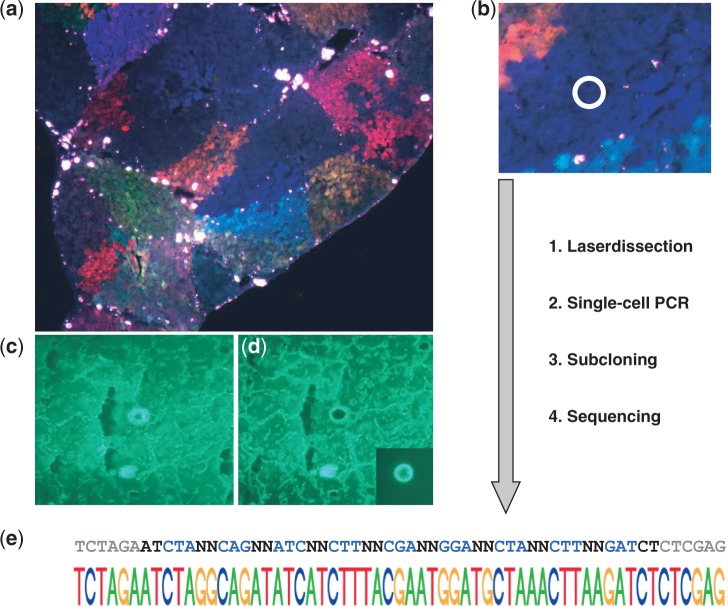
Analyzing single cells from regenerated liver tissue. (**a**) LeGO-C2-BC16 (red), LeGO-V2-BC16 (yellow-green) and LeGO-Cer2-BC16 (cyan-blue) were used for transduction of primary murine hepatocytes. Transduced cells were transplanted intrasplenically into hemizygous uPA/SCID mice. Liver sections taken 4 weeks post-transplantation show a regeneration of the liver with RGB-marked patches. (**b**) Schematic representation of the experimental procedure: laser dissection of single cells from cryosections of the regenerated liver, single-cell PCR, subcloning of the obtained PCR fragment and sequencing of the obtained clones. (**c**, **d**) Laser dissection of one single cell from the liver section. (**e**) Sequencing of bacterial clones revealed a single barcode with the LeGO-Cer2-BC16 signature encoding for the Cerulean fluorescence protein.

To assess the applicability of combined RGB marking/barcoding *in vivo*, we made use of our well-established liver regeneration model. Therefore, we simultaneously transduced primary mouse hepatocytes with LeGO-C2-BC16 (red), LeGO-V2-BC16 (yellow-green) and LeGO-Cer2-BC16 (cyan-blue) at equal MOI using a short-term transduction protocol (7). RGB-marked hepatocytes were transplanted intrasplenically in 4-week-old hemizygous urokinase-type plasminogen-activator immunodeficient uPA/SCID mice (24). In these mice, expression of the uPA transgene in the liver leads to hepatocyte destruction facilitating engraftment and proliferation of transplanted normal hepatocytes. As previously reported (7), engraftment of RGB-marked hepatocytes results in patches of regeneration that are marked by specific color hues (Figure 2a). We now asked whether this *in situ* setting also allows for the correlation of a given color with its corresponding barcode (Figure 2b). To answer this question, we isolated single cells out of patches of engrafted, RGB-marked hepatocytes using laser microdissection (Figure 2c and d). We performed single-cell PCR on these cells and were able to identify unique barcode sequences (Figure 2e). As in the *in vitro* setting (Figure 1), the identified barcode contained the vector signatures corresponding to the observed RGB colors.

### Tracking ΔTrkA-induced leukemia in mice based on barcodes

**Figure 3.**
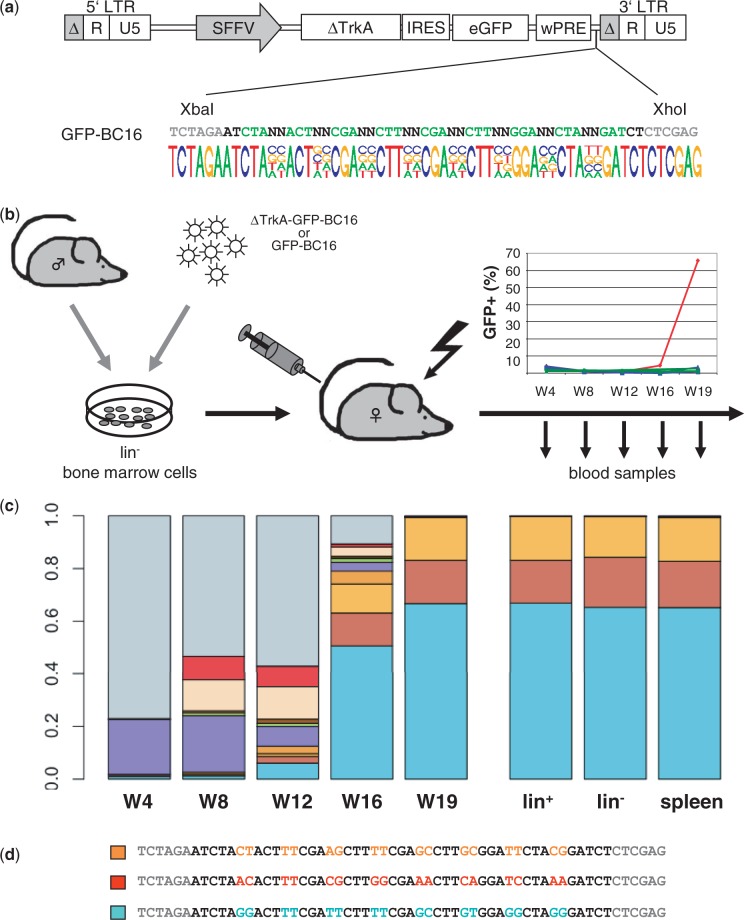
Barcodes for tracking leukemia. (**a**) Schematic representation of vector ΔTrkA-LeGO-iG2-BC16 co-expressing the oncogene ΔTrkA and eGFP and equipped with a GFP-BC16 barcode library. The barcode library consisting of >700 000 different plasmids showed an equal distribution of the randomized nucleotides as evident from Illumina sequencing (>26 Mio reads) on 10^10^ plasmids (illustrated in the frequency plot). (**b**) Viral supernatant of the ΔTrkA-LeGO-iG2-BC16 plasmid library was used to transduce syngeneic lineage-negative bone marrow cells from male donors. Transduced cells were transplanted into lethally irradiated female recipient mice (*n* = 4). Control mice (*n* = 4) were transplanted with a barcoded eGFP marking vector (LeGO-G2-BC16). During follow-up, blood was taken every 4 weeks from transplanted mice. One mouse developed full-blown leukemia after 19 weeks as evidenced by the high proportion of eGFP-positive cells in the blood. All other mice showed stable eGFP counts in the peripheral blood during follow-up analysis. (**c**) Frequency analysis (stacked box plot) for barcodes found in leukemia samples by NGS of DNA from blood, spleen and bone marrow cells. The 10 most abundant barcodes were given individual colors, all other barcode sequences are summarized by gray boxes. (**d**) Sequences of the three leukemia-contributing barcodes, wobble bases are marked in accordance with the color of the respective box plot in (c).

To address the usefulness of barcoding to track clonal outgrowth of malignant cells, we made use of the model oncogene ΔTrkA previously shown to induce different types of leukemia in a murine bone marrow transplantation model (25,26) (Figure 3b). Eco-*Env* pseudotyped barcoded lentiviral vectors encoding ΔTrkA (in conjunction with eGFP) (Figure 3a) could successfully be produced, albeit at relatively low titers (5.4 × 10^5^/ml); for the barcoded eGFP-only control vector (Supplementary Figure S1a), the titer was 6.4 × 10^6^/ml. Transduction of lineage-depleted donor bone marrow cells resulted in comparatively low numbers of transgenic cells (1.7% for ΔTrkA-eGFP and 3.1% for eGFP-control) as assessed by FACS analysis for eGFP. Four recipient mice were transplanted with 300 000 lin^−^ bone marrow cells containing ∼5000 ΔTrkA-positive barcoded cells (and ∼9000 for the control eGFP animals).

Nineteen weeks after transplantation, one mouse died of acute leukemia. At this point, all remaining animals were humanely killed, and peripheral blood, spleen and bone marrow were analyzed for eGFP expression (Supplementary Figure S1b). For the leukemic mouse, FACS analysis revealed that the majority of blood cells expressed eGFP (peripheral blood: 66%, spleen: 73%, bone marrow—lin^−^ compartment: 84%, lin^+^ compartment: 89%). Interestingly, malignant cells were positive for the B-cell marker B220 (Supplementary Figure S3a). This phenotype was confirmed by transplantation of the leukemia into secondary recipients (Supplementary Figure S3b).

In one further animal of the ΔTrkA group, an increase in the percentage of transgenic cells indicating a preleukemic stage was observed in all hematopoietic organs, particularly in the bone marrow (>25% in the lin^+^ fraction; Supplementary Figure S1b). The two other animals were healthy at the time of analysis. Also, none of the four control animals showed increased numbers of eGFP-positive cells.

To assess clonal composition of the observed leukemia, we performed NGS of cells obtained from the hematopoietic organs of the diseased animal. We found three strongly dominant barcodes in leukemic cells (Figure 3c and d). To verify whether these barcodes represented one, two or three clones, we estimated vector-copy numbers in leukemic cells by ddPCR. Notably, ddPCR confirmed the presence of three vector insertions in all leukemic cells. Therefore, we concluded that, despite the initially low gene transfer efficiency, the diseased mouse developed a monoclonal leukemia containing three vector copies that corresponded to three different barcodes.

We next performed LM-PCR to identify vector-integration sites in the genome of leukemic cells. As expected, we were able to retrieve three insertion sites located on chromosomes 10, 17 and 19. Each integration site could be linked to one corresponding barcode by PCR using the BC-PCR-Seq as a forward primer and an integration-specific reverse primer (Supplementary Table S1) followed by Sanger sequencing of the PCR product (Supplementary Figure S2). Interestingly, the integration on chromosome 19 was located in proximity to Tle4—a corepressor of Pax5. Therefore, it is tempting to speculate that insertional mutagenesis has contributed to the specific B220^+^-phenotype of the observed leukemia.

To ultimately prove monoclonality of the observed leukemia, we transplanted spleen cells from the diseased animal into a second cohort of mice (*n* = 10, 1 × 10^6^ cells per animal). All secondary recipients rapidly developed leukemia; two died on day 13 and the others had to be killed on day 14. Leukemias available for analysis (*n* = 8) uniformly showed the B-cell phenotype (B220-positivity) and were positive for all three insertion sites (Supplementary Figure S4).

## DISCUSSION

We have described a novel approach for the marking of individual cells, which comprises the virtues of two recently established marking techniques, namely, RGB marking (7) and DNA barcoding (13–15). While the fluorescence-based RGB marking allows phenotypic distinction of different cell clones *in situ* (7), molecular marking based on unique barcode sequences enables the robust and long-term follow-up even if the expression of fluorescence genes were diminished (13–16).

Using three different models, we have provided proof-of-principle for the usefulness of the system. We first applied our marking technique *in vitro* on 293 T cells previously shown to facilitate efficient RGB marking (*7*). We picked live RGB-marked 293 T cells of the same color hues and demonstrated clonal identity based on sequence analysis of the barcodes amplified by single-cell PCR. Although the experiment might seem trivial on the first glance, it has important implications. In fact, expression of the same color hue does not *per se* prove clonal identity and vice versa. Obviously, the number of distinguishable colors after RGB marking is limited for a number of reasons (*7*). Moreover, color hues are expected to slightly differ even within a given clone, for instance, due to different phases of cell cycle or metabolic activity of individual cells of that clone. Therefore, molecular analysis, e.g. of vector-insertion sites (7), may be necessary to confirm clonal identity of two different cells apparently belonging to the same clone. However, cloning of insertion sites is cumbersome and sometimes even impossible depending on the actual integration locus (8). On the contrary, PCR amplification and sequencing of barcodes are straightforward and independent of the specific vector-insertion site.

In the second approach, we exploited our barcoded vectors to RGB mark primary mouse hepatocytes that were subsequently transplanted into livers of immunodeficient uPA/SCID mice, a well-established liver-regeneration model (24). As previously shown (7), engraftment of RGB-marked hepatocytes resulted in a patched liver architecture, in which individual clones contributing to regeneration were distinguishable based on their color hues. In this work, single cells from those regeneration areas were picked using laser microdissection and analyzed by barcode amplification and sequencing. Thus, we were able to demonstrate that molecular analysis of clonal identity as described above is also applicable for fixed cells. This might be important in various experimental settings, not the least, as fixed cells often display lost or altered fluorescence characteristics (27). From a practical point it needs to be added that, dependent on the thickness of the tissue slice, it might be necessary to isolate more than one cell to make sure that the cut sample contains an entire nucleus.

In the liver regeneration model used here, barcoding combined with NGS may be applied to quantitatively assess clonal reconstitution. One might argue that the necessity to introduce several vector copies (mean of three) to achieve efficient RGB marking could impede clonal tracking. However, to achieve marking of a high proportion of any cells of interest, multiple vector integrations have invariably to be accepted (28), at least if sorting of marked cells does not represent an option (as with primary hepatocytes that can be cultured *ex vivo* only short-term). Quantitative barcode analysis in such case needs to be combined with vector-copy determination, e.g. by qPCR or ddPCR. The additional information available from the RGB marking and the use of three different barcode vectors with individual signatures can be expected to significantly ease this task. Also, complexity of analysis might be reduced by using arrayed barcodes (29).

The possibility to confirm clonal identity of RGB-marked cells is even more important for a variety of other *in vivo* settings, namely, those potentially associated with losses or changes of transgene expression resulting in ‘new’ mixed colors. Relevant applications include RGB marking of stem cells (27), which may change expression patterns on differentiation, but also RGB marking of tumor cells, which may lose expression of (some) colors due to chromosomal instability or epigenetic changes. In both settings, the opportunity to address clonal identity is crucial for the correct interpretation of results. Also, the detection of rare events such as disseminated tumor cells and/or circulating tumor cells (30) based on fluorescence is still challenging. Here, the additional introduction of barcodes in combination with fluorescent marking is expected to further improve sensitivity and help overcoming potential issues with a loss of transgene expression.

We finally assessed the benefit of barcoded vectors in the setting of clonal leukemia development. To do so, we made use of an established murine transplantation model based on the expression of the oncogene ΔTrkA in hematopoietic stem and progenitor cells (26). We found that barcoding not only facilitates clonal analysis of hematopoietic reconstitution (14,15,29,31,32), but as well allows to monitor outgrowth of leukemic cells. Based on our proof-of-concept experiment, we suggest that analysis of clonal evolution of blood (and other) malignancies represents a promising application of cell marking with barcoded vectors. To this end, we have cloned a set of barcoded RGB vectors for the expression of different (onco-) genes in conjunction with the fluorescent markers (data not shown).

In our study, we also improved the barcode design introduced by Gerrits *et al.* (14). Using the fixed nucleotides located between the ‘wobbled’ bases, we introduced a vector-specific (here color-specific) signature in the barcode. This enabled us to not only identify the barcodes in a given cell, but also to molecularly assess the combinations of RGB colors present in that cell. Thus, we could correlate the actual colors seen in the microscope with the theoretically expected ones based on the additive color model. Whereas actual and theoretically expected colors perfectly matched in our experiments, the specific color signatures introduced in the barcodes would also allow to identify cells that have lost expression of one or the other transgene. Moreover, the suggested barcode design opens up a number of interesting applications. In fact, barcodes with individual signatures allow to differentially label and subsequently follow-up distinct cell populations (e.g. stem cells isolated using alternative markers, cultured under different conditions or transduced with different genes) to assess both inter- and intra-clonal heterogeneity in competitive transplantation experiments. Therefore, we propose that these novel barcodes will be useful for a variety of experimental settings in regenerative medicine, particularly hematopoietic stem cell transplantation, but also to study clonal evolution of malignant diseases.

In conclusion, we have successfully combined two cutting-edge technologies to unambiguously mark and follow-up single-cell-derived clones at high levels of resolution and accessibility. The novel marking approach proposed here will be highly instrumental for studies aiming toward the identification of clonal growth patterns in regenerative medicine, but also cancer research.

## SUPPLEMENTARY DATA

Supplementary Data are available at NAR Online.

## FUNDING

The German Research Foundation (DFG) [FE568/11-1 to B.F., SFB841 to B.F. and M.D., RO3500/1-2 to I.R. and SFB655 to A.D.]. The German Cancer Aid (Deutsche Krebshilfe) [110619 to K.C., B.F., I.G. and I.R.]; Young investigator grant within the FFM program of the UMC Hamburg-Eppendorf [NWF-12/02 To K.C.]. Funding for open access charge: Deutsche Forschungsgemeinschaft.

*Conflict of interest*: Author Michael Forgber is an employee of ALS Automated Lab Solutions GmbH (Jena, Germany), the company manufacturing the CellCelector device used in this study. All other authors declare that they have no conflict of interest associated with the presented data.

## Supplementary Material

Supplementary Data
