# Supplementary material for: Multiplexing clonality: combining RGB marking and genetic barcoding
Source: Nucleic Acids Res. 2014 Jan 28;42(7):e56. doi: 10.1093/nar/gku081 (PMC3985654; doi:10.1093/nar/gku081)
Supplement: Supplementary Data [file supp_42_7_e56__index.html]

Multiplexing clonality: combining RGB marking and genetic barcoding — Multiplexing clonality: combining RGB marking and genetic barcoding — Supplementary Data 

# Multiplexing clonality: combining RGB marking and genetic barcoding

## Supplementary Data

files

**Files in this Data Supplement:**

- Supplementary Data - pdf file
