# Supplementary material for: Multiplexing clonality: combining RGB marking and genetic barcoding
Source: Nucleic Acids Res. 2014 Jan 28;42(7):e56. doi: 10.1093/nar/gku081 (PMC3985654; doi:10.1093/nar/gku081)
Supplement: Supplementary Data [file supp_gku081_nar-03654-met-h-2013-File005.pdf]

Supplementary table 1: Sequences

Barcode Oligos

Name	Sequence (5'-3')
mCherry-BC16 FW	CTAGAATCTANNCTANNCAGNNCTTNNCGANNCTANNCTTNNGGANNGATCTC
mCherry- BC16 RV	TCGAGAGATCNNTCCNNAAGNNTAGNNTCGNNAAGNNCTGNNTAGNNTAGATT
Venus- BC16 FW	CTAGAATCTANNCACNNAGANNCTTNNCGANNCTANNGGANNCTTNNGATCTC
Venus- BC16 RV	TCGAGAGATCNNAAGNNTCCNNTAGNNTCGNNAAGNNTCTNNGTGNN TAGATT
Cerulean- BC16 FW	CTAGAATCTANNCAGNNATCNNCTTNNCGANNGGANNCTANNCTTNNGATCTC
Cerulean- BC16 RV	TCGAGAGATCNNAAGNNTAGNNTCCNNTCGNNAAGNNGATNNCTGNNTAGATT
GFP- BC16 FW	CTAGAATCTANNACTNNCGANNCTTNNCGANNCTTNNGGANNCTANNGATCTC
GFP- BC16 RV	TCGAGAGATCNNTAGNNTCCNNAAGNNTCGNNAAGNNTCGNNAGTNNTAGATT

PCR and Sequencing primers

Name	Sequence (5'-3')
p90	GGACGTCCTTCTGCTACGT
p91	GAGCTCCCAGGCTCAGA
BC-PCR-FW	CATCGATACCGTCGACCTC
BC-PCR-RV_neu	GCTAAGATCTACAGTCTCGAGAGATC
BC-PCR-Seq	ACAGCAGCTACCAATGCTGA
Ill2_Tail-complete	GTGACTGGAGTTCAGACGTGTGCTCTTCCGATCTCATCGATACCGTCGACCTC
Ill1-Tail12	ACACTCTTTCCCTACACGACGCTCTTCCGATCTTCTCGAGAGATC
Universal primer (P34)	AATGATACGGCGACCACCGAGATCTACACTCTTTCCCTACACGACGCTCTTCCGATCT
Indexing primer	CAAGCAGAAGACGGCATACGAGAT XXXXXX GTGACTGGAGTTC
Bridging oligonucleotide	GTGACTGGAGTTCAGACGTGTGCTCTTCCGATC
Integration1-rv	GTAGAGGCAAGTGGGGAAGA
Integration2-rv	CCTGGGTGGTGAAGAGAAGA
Integration3-rv	AGAAACTGCCTCCTGGGATT

digital droplet PCR

Name	Sequence (5'-3')
FP-dPCR-fw	CAGGAGCGCACCATCTTCTT
FP-dPCR-rv	AGGGTGTCGCCCTCGAAC
FP-probe	CTACAAGACCCGCGCCGAGGTGA
mEpo-fw	GCAGGCGGGGTCGCTACTC
mEpo-rv	CGCCTGTGCAGATCCGATAA
mEpo-probe	TTCTGAGGCGCCACTTTTGCAAGACC

Supplementary Figure 1

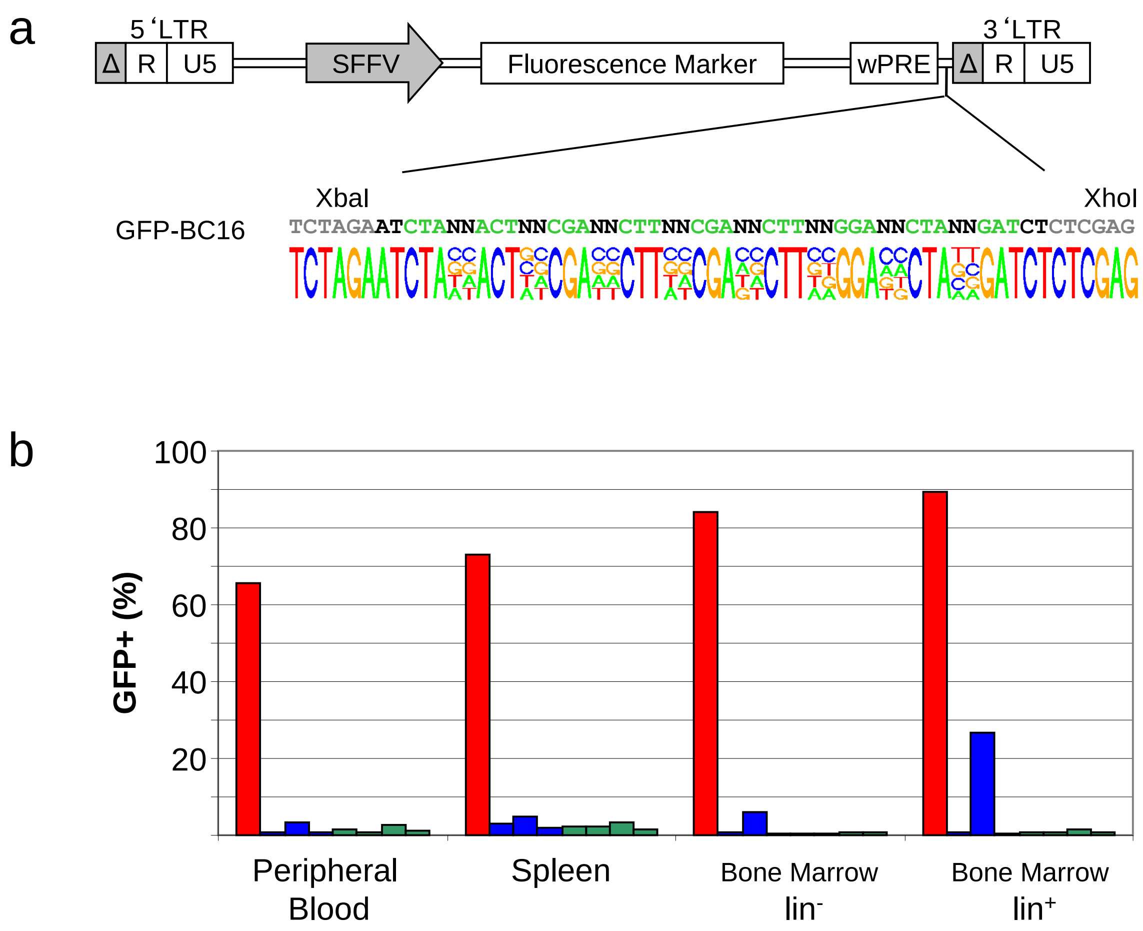

Supplementary Figure 1: eGFP-control vector and FACS-analysis at final analysis

(**a**) Schematic representation of vector LeGO-G2-BC16 equipped with a GFP-BC16 barcode library. The barcode library consisting of approx. 600.000 different plasmids showed an equal distribution of the randomized nucleotides as evident from Illumina sequencing (>26 Mio reads) on 10^{10} plasmids, as shown in the frequency plot. (**b**) FACS-analysis for eGFP-expression of haematopoietic organs at final analysis of the mouse transplantation experiment shown in Figure 3b. The animal with the described leukemic phenotype is shown in red. The other 3 animals which received the ΔTrkA-transduced cells are shown in blue. eGFP-control animals are shown in green.

Supplementary Figure 2

a

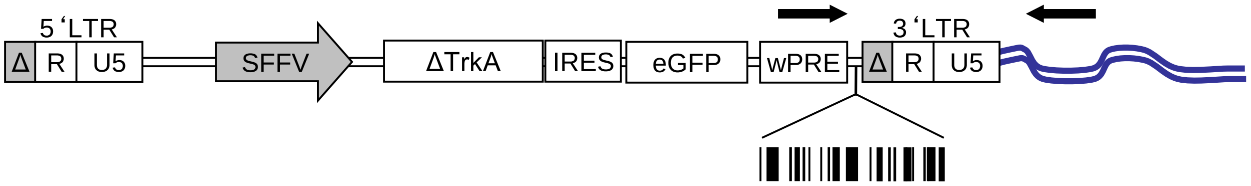

b

Integration 1, Chromosome 10:
CCATCTAGAATCTACTACTTTCGAAGCTTTTCGAGCCTTGCGGATTCTACGGATCTCTCGAGACT
GTAGATCTTAGCCACTTTTTAAAAGAAAAGGGGGG[3 ‘LongTerminalRepeat]CAACAATC
AGGAGAGAAAAAAATCAATAAAACCAAAAAAAAAGTTCAGTTATTCTGACCTAAGTATCTGTGGC
ACTGGATGACAATGACTGGAAAGCCAGCAGCCATGACCTAGGGCTCTGGGAAGGGATGTGATGCA
GCCAAGGGAGCCTGAGAGCCTGAGGGCACAGATAGGAAAGAGGGAGGGATTGCATTTAGATGGAT
GCTACGATCATACAG

Integration 2, Chromosome 17:
CCATCTAGAATCTAACACTTTCGACGCTTGGCGAAACTTCAGGATCCTAAAGATCTCTCGAGACT
GTAGATCTTAGCCACTTTTTAAAAGAAAAGGGGGG[3 ‘LongTerminalRepeat]CATAGAGA
GTATTCTACTACATACAAAGGGAATATTATGATATTAGTCTCAGTGCAATTATCTAAAAACAATT
GAAATGAAATAGGATCTGTCGAAACCAATTCCATGTCCTCTTTCCACTGTCAGTTGAGCCTGCCC
ACGCCCAAGGCTTCCTCATTCTCTCTAACATGCCTGGATCCTTTGCTACACCTTCTT

Integration 3, Chromosome 19:
CCATCTAGAATCTAGGACTTTCGATTCTTTTCGAGCCTTGTGGAGGCTAGGGATCTCTCGAGACT
GTAGATCTTAGCCACTTTTTAAAAGAAAAGGGGGG[3 ‘LongTerminalRepeat]TAGACTAG
GGACAAGTTTACTCCTGTGTAGTAAATCTAAATGGTGGAAATTTAACTTAAGATTATTTTCCCAC
ACAAACTGTCTGAGGACCCGATATTGTGCCAGTTAATCC

Supplementary Figure 2:

(**a**) Scheme of proviral integration site and primer locations. (**b**) Sequences of obtained barcodes in conjunction with their integration site. Wobble bases are colored in the same manner as in Figure 3.

Supplementary Figure 3

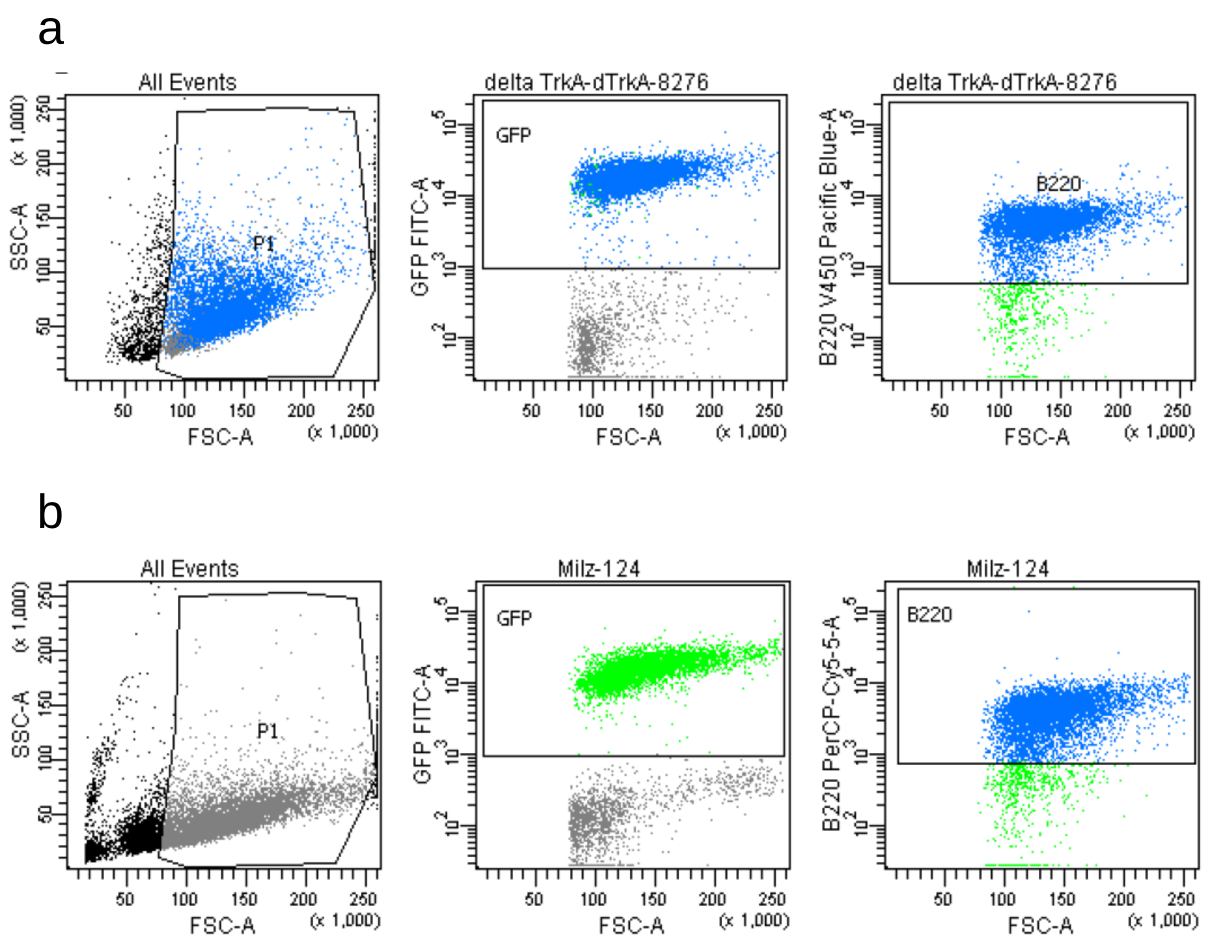

Supplementary Figure 3: FACS analysis of spleen cells from leukemic mice.

(**a**) Flow-cytometry analysis of eGFP and B220 expression in leukemic cells from the spleen of the diseased animal in the first mouse cohort. B220-positive cells were stained with a V450-coupled antibody. (**b**) Analogous FACS analysis on spleen cells from one representative animal from the second cohort (after serial transplantation of the leukemia). B220-positive cells were stained with a PerCP-Cy5.5-coupled antibody. In both samples, approx. 90% of leukemic cells co-express B220 and eGFP.

Supplementary Figure 4

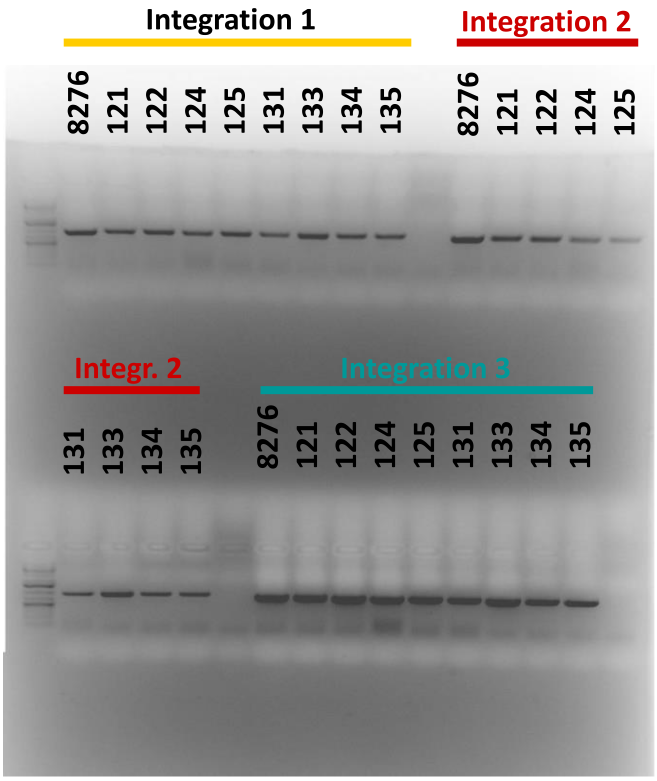

Supplementary Figure 4: Confirmation of the presence of all three vector integrations in leukemic mice

PCR was performed with a vector-specific forward primer (BC-PCR-Seq) and integration-specific reverse primers for each integration on DNA from spleen samples of the diseased mouse from the first cohort (#8276) and all eight animals available foe analysis from the second cohort (#121-122, 124-125, 131, 133-135).
